# Effectiveness of convalescent plasma therapy in eight non-intubated coronavirus disease 2019 patients in Indonesia: a case series

**DOI:** 10.1186/s13256-021-03059-y

**Published:** 2021-11-23

**Authors:** Theresia Monica Rahardjo, Elizabeth Yogipranata, Ardian Hediyanto Naswan, Fitri Rahayu Sari, Fajar Budiono, Hernawati Permatasari, C. H. R. Driantik Chuntari

**Affiliations:** 1 Faculty of Medicine, Maranatha Christian University, Suria Sumantri 65, Bandung, West Java 40164 Indonesia; 2 Primaya Hospital, Tangerang, Indonesia; 3 Present Address: Eka Hospital, Pekanbaru, Indonesia; 4 Present Address: Eka Hospital, Tangerang, Indonesia

**Keywords:** Convalescent plasma therapy, Non-intubated, Case series

## Abstract

**Background:**

Severe acute respiratory syndrome coronavirus 2, the cause of coronavirus disease 2019, has become a global pandemic. Currently, there is no definitive treatment for coronavirus disease 2019. Convalescent plasma therapy has become a potential specific curative method, while vaccines as protection modalities require further work.

**Case presentation:**

Eight non-intubated Indonesian patients, ages ranging from 40 to 74 years old, with coronavirus disease 2019 confirmed by viral Ribonucleid Acid (RNA) real-time polymerase chain reaction tests were included. Four patients were administered two doses of 200 mL convalescent plasma, and the other four patients were administered one dose of convalescent plasma with an antibody titer of 1:320, within the first 14 days since symptoms occurred. The median times from illness onset to convalescent plasma therapy and from the first day of hospital admission to convalescent plasma therapy were 13 and 6.5 days, respectively. All patients showed improvements in clinical symptoms, laboratory parameters, thorax imaging, negative conversion of polymerase chain reaction results, and decreased oxygen supplementation within 1 week after convalescent plasma therapy. Patients with two convalescent plasma doses tended to have faster recovery than those with one convalescent plasma dose. No severe adverse effects were observed in any patient.

**Conclusion:**

This is the first case series in Indonesia showing that convalescent plasma therapy is safe and well tolerated and that early convalescent plasma therapy before the patient is intubated could potentially prevent disease progression, increase the recovery rate, and shorten the inpatient time of stay.

## Background

The coronavirus disease 2019 (COVID-19) pandemic has been ongoing for more than 1 year since the first case emerged in Wuhan, China, in December 2019. The disease spread rapidly, and within 3 months, it was defined as a pandemic by the World Health Organization (WHO) on 11 March 2020. The first two cases in Indonesia were confirmed on 2 March 2020, and subsequently, the number of confirmed positive cases rose to more than 1 million in early 2021 [1].

Currently, there have been no approved antiviral agents targeting the virus. Furthermore, the use of corticosteroid agents for COVID-19 patients is controversial because immune suppression causes delayed viral clearance and some complications. Vaccines have already been produced and have different targets to prevent or protect against the disease. This situation makes convalescent plasma (CP) therapy (CPT) a promising therapy for patients with COVID-19 [2].

Sources for CPT in Indonesia can be identified in two ways: first by patient self-identification and second by research studies, as initiated in April 2020. This treatment modality was already known more than 100 years ago when the Spanish flu was rampant, and this method showed the ability to reduce the mortality rate among patients. Subsequently, CPT was used to treat the severe acute respiratory syndrome (SARS), Middle East respiratory syndrome (MERS), and 2009 H1N1 pandemics with significant efficacy and safety. Many studies, from case reports to meta-analyses, have shown the effectiveness of CPT. This study is the first case series of CPT effectiveness in eight non-intubated COVID-19 Indonesian patients at a private hospital in Indonesia [3, 4].

## Case presentation

Eight non-intubated COVID-19 patients, including five men and three women aged 40–74 years, were included in this case series and received CPT. The median age was 56.25 years. The median time from onset of illness to CPT was 13 days, and from the first day of hospitalization to CPT was 6.5 days. All patients originated from the Tangerang area, and none of them traveled abroad.

All patients had severe COVID-19. The most common symptoms were fever and dyspnea in seven patients, cough in five patients, nausea in three patients, and diarrhea in two patients. Six patients had comorbidities, including diabetes mellitus, hypertension, cardiovascular disease, respiratory disease, and blood disorders. The patient characteristics are listed in Table 1.

**Table 1:** Clinical characteristics of patients receiving Convalescent Plasma Therapy

No.	Patient	Sex	Age (years)	Stage of disease	Days from symptom onset to admission	Days from symptom onset to CPT	Site of infection	Main symptoms	Comorbidities
1	SUN	M	50	Severe COVID-19	5	9	Lung	Fever, cough, dyspnea	Hypertension, diabetes mellitus type 2, anxiety disorder
2	HSD	M	67	Severe COVID-19	7	16	Lung	Fever, cough, nausea, dyspnea	CAD post PCI, diabetes mellitus type 2, hypertension
3	YHA	F	50	Severe COVID-19	7	13	Lung	Dyspnea, fever, cough, diarrhea	None
4	AMH	F	45	Severe COVID-19	4	10	Lung	Dyspnea, fever, cough, myalgia	Diabetes mellitus type 2
5	ERN	M	55	Severe COVID-19	7	15	Lung	Dyspnea, cough, fever	Diabetes mellitus type 2
6	DSO	M	40	Severe COVID-19	3	11	Lung	Dyspnea, fever, fatigue, nausea	None
7	RMD	M	51	Severe COVID-19	5	11	Lung	Fever, diarrhea	Polycythemia vera
8	HUM	F	74	Severe COVID-19	14	19	Lung	Nausea, general weakness, dyspnea	Diabetes mellitus type 2, arrhythmia (frequent VES)

CAD: Coronary Artery Disease, PCI: Percutaneous Coronary intervention, VES: Ventricular Extra Systole, CPT: Convalescent Plasma Therapy, CAD: Coronary Artery Disease, PCI: Percutaneous Coronary intervention, VES: Ventricular Extra Systole

Each patient received standard therapy, including antiviral, antibiotic, and corticosteroid therapy with 2.5–5 mg dexamethasone intravenously administered one to three times daily. All patients received oxygen support and showed bilateral suprahilar, perihilar, and paracardial pulmonary parenchymal consolidation in thorax photo, with one patient having a thorax computerized tomography (CT) scan, which showed ground-glass opacity (GGO). Nasopharynx real-time polymerase chain reaction (RT-PCR) swab yielded positive results with cycle threshold (CT) values varying between 21.11% and 33.13% for lung involvement. The standard treatment for all patients is presented in Table 2.

**Table 2:** Standard treatment of patients receiving Convalescent Plasma Therapy

No.	Antiviral	Antibiotic	Corticosteroid/Other	Oxygen support	
				Before CPT	After CPT
1	Oseltamivir 2 × 75 mg (PO)	Azithromycin 1 × 500 mg (PO) Meropenem 3 × 1 g (IV) Cefepime 2 × 1 g (IV)	Dexamethasone 1 × 2.5 mg (IV) Tocilizumab 400 mg (IV)	NRM 15 L/min	NC 4 L/minute 5 days after CPT
2	Lopinavir 2 × 2 tab (PO) Isoprinosine 4 × 500 mg (PO) Oseltamivir 2 × 75 mg (PO)	Azithromycin 1 × 500 mg (IV) Levofloxacin 1 × 750 mg (IV)	Dexamethasone 3 × 5 mg (IV)	Non Rebreathing Mask 15 L/minute	NC 3 L/minute 3 days after CPT
3	Methisoprinol 4 × 500 mg (PO)	Meropenem 3 × 1 g (IV) Azithromycin 1 × 500 mg (PO)	Dexamethasone 2 × 5 mg (PO)	Non Rebreathing Mask 15 L/minute	NC 3 L/minute 1 day after CPT
4	Favipiravir 2 × 800 mg day 1 and 2 × 600 mg days 2–6 (PO) Intravenous immunoglobuline 1 × 25 g for 5 days (IV)	Levofloxacin 1 × 500 mg (IV) Azithromycin 1 × 500 mg (PO) Meropenem 3 × 1 g (IV)	Dexamethasone 3 × 5 mg (PO) Tocilizumab 400 mg (IV)	HFNC FiO_2_ 70% flow 40 L/minute	High Flow Nasal Cannula FiO_2_ 40% flow 35 L/minute 5 days after CPT
5	Lopinavir 2 × 2 tab Methisoprinol 3 × 1 (PO) Favipiravir 2 × 1600 mg day 1, 2 × 600 mg day 2–5 (PO)	Levofloxacin 1 × 750 mg (IV) Meropenem 3 × 1 g (IV)	Dexamethasone 3 × 5 mg (IV)	Non Rebreathing Mask 15 lpm	Room air 3 days after CPT
6	Methisoprinol 3 × 500 mg (PO) Favipiravir 2 × 1600 mg day 1, 2 × 600 mg days 2–5 (PO)	Azithromycin 1 × 500 mg (PO)	Dexamethasone 3 × 5 mg (IV)	Simple mask 8 L/minute	NC 3 L/minute 2 days after CPT
7	Favipiravir 2 × 600 mg (PO)	Levofloxacin 1 × 750 mg (IV) Ceftriaxone 1 × 2 g (IV) Azithromycin 1 × 500 mg (PO) Meropenem 3 × 1 g (IV)	Dexamethasone 1 × 2.5 mg (IV) Tocilizumab 400 mg (IV)	NC 3 L/minute	Room air 8 days after CPT
8	Favipiravir 2 × 1600 mg day 1, 2 × 600 mg days 2–5 (PO)	Levofloxacin 1 × 750 mg (IV) Meropenem 3 × 1 g (IV) Ceftazidime 3 × 1 g (IV)	Dexamethasone 2 × 5 mg (IV)	High Flow Nasal Cannula FiO_2_ 90% flow 50 L/minute	Non Rebreathing Mask 15 L/minute 6 days after CPT

CPT: Convalescent plasma therapy, NC: Nasal Cannula, NRM: Non Rebreathing Mask, IV: Intravenous, PO: Peroral

Four patients received two doses of convalescent plasma (CP), and another four patients received one dose of CP. Each dose contained 200 mL of CP and was given over 4 hours with continuous observation and monitoring. The two-dose regimens were administered within 1 week [4, 5]. All CPs were processed by the Indonesian Red Cross (PMI), a humanitarian organization and a member of International Federation of Red Cross. Each CP contained a 1:320 antibody level against SARS-CoV-2 [5, 6].

All symptoms in the eight non-intubated patients, especially fever, dyspnea, and cough, were reduced or disappeared within 1–7 days after CP transfusion. Prior to CPT, four patients used a nonrebreathing mask (NRM) at 15 L/minute, one patient used a simple mask (SM) at 8 L/minute, one patient used a nasal cannula (NC) at 3 L/minute, and two patients used a high-flow nasal cannula (HFNC). Following CP transfusion, all patients felt better and showed improvement, with a reduction in oxygen supplementation, which gradually started 1 day after CPT. Two patients discontinued HFNC therapy and shifted to a NRM within a week. Two patients shifted from a NRM to a NC within 3 days. One patient shifted from 15 L/minute to room air 3 days after CPT.

Ideally, pulmonary function can be assessed from oxygen saturation measured from the central vein (SaO_2_) and the ratio of SaO_2_ to oxygen fraction (PF ratio), but there was limited capacity for this monitoring, as for chest radiography and laboratory tests. In this case series, noninvasive oxygen saturation (pulse oximetry) was used as an alternative way to monitor pulmonary function, accompanied by respiratory rate and oxygen supplementation monitoring. All patients showed an increase in oxygen saturation from days 1 to 7 after CPT. A comparison of respiration parameter before and after CPT is presented in Table 3.

**Table 3:** Comparison of parameters before and after Convalescent Plasma Therapy

Parameter	CPT	Time	Patient 1	Patient 2	Patient 3	Patient 4	Patient 5	Patient 6	Patient 7	Patient 8
Respiration	Before		19–20	28	28–33	36	27–30	40	17–18	22–24
	After	Day 1	20–23	16–20	20–22	30	20–22	28	18–20	20–22
		Day 3	16	16–18	18–20	15–17	19–20	18–20	18–20	20–22
		Day 5	24	16–18	18–20	22–27	19–20	18–19	18–20	18–20
		Day 7	16–18	16–18	18–20	20–24	16–18	18–19	18–20	18–20
O_2_ support	Before		NRM 12 L/minute	NRM 15 L/minute	NRM 15 L/minute	HFNC FiO_2_ 70% flow 40 L/minute	NC 4 L/minute	NRM 15 L/minute	NC 3 L/minute	HFNC FiO_2_ 95% flow 60 L/minute
	After	Day 1	NRM 15 L/minute	NRM 15 L/minute	NC 3 L/minute	HFNC FiO_2_ 70% flow 40 L/minute	NC 4 L/minute	NRM 10 L/minute	NC 3 L/minute	HFNC FiO_2_ 90% flow 60 L/minute
		Day 3	NRM 15 L/minute	NC 3 L/minute	NC 3 L/minute	HFNC FiO_2_ 70% flow 50 L/minute	NC 4 L/minute	NC 4 L/minute	NC 3 L/minute	HFNC FiO_2_ 50% flow 35 L/minute
		Day 5	NC 4 L/minute	Room air	NC 3 L/minute	HFNC FiO_2_ 40% flow 35 L/minute	NC 3 L/minute	NC 4 L/minute	NC 3 L/minute	HFNC FiO_2_ 35% flow 30 L/minute
		Day 7	NC 4 L/minute	Room air	Room air	NC 4 L/minute	Room air	Room air	NC 3 L/minute	NRM 15 L/minute
O_2_ saturation	Before		96–97	94	97–98	90	94–96	90	95–98	90–93
	After	Day 1	94–96	97	97–98	95–99	96	90–94	97–98	96–99
		Day 3	98	98	98–99	97–98	93–97	96	99	97–98
		Day 5	97–98	98–99	98–99	95–98	97–98	97–98	99	97–98
		Day 7	99	98–99	98–99	95–97	98–99	98	99	97–98
Thorax imaging	Before		Bilateral infiltrate	Bilateral infiltrate	Bilateral infiltrate	No specific process	Bilateral infiltrate	–	Bilateral infiltrate	Bilateral infiltrate
	After	Day 1	–	–	–	Bilateral infiltrate	–	–	–	–
		Day 3	Infiltrate reduction	–	–	–	–	–	–	–
		Day 5	–	–	–	Infiltrate reduction	Bilateral infiltrate	–	Bilateral infiltrate	–
		Day 7	Infiltrate reduction	Infiltrate reduction	Infiltrate reduction	–	Infiltrate reduction	Paracardial infiltrate	Infiltrate reduction	Infiltrate reduction

CPT: Convalescent plasma therapy, NC: Nasal cannula, NRM: Non rebreathing mask, HFNC: High flow nasal cannula

**Fig. 1:**
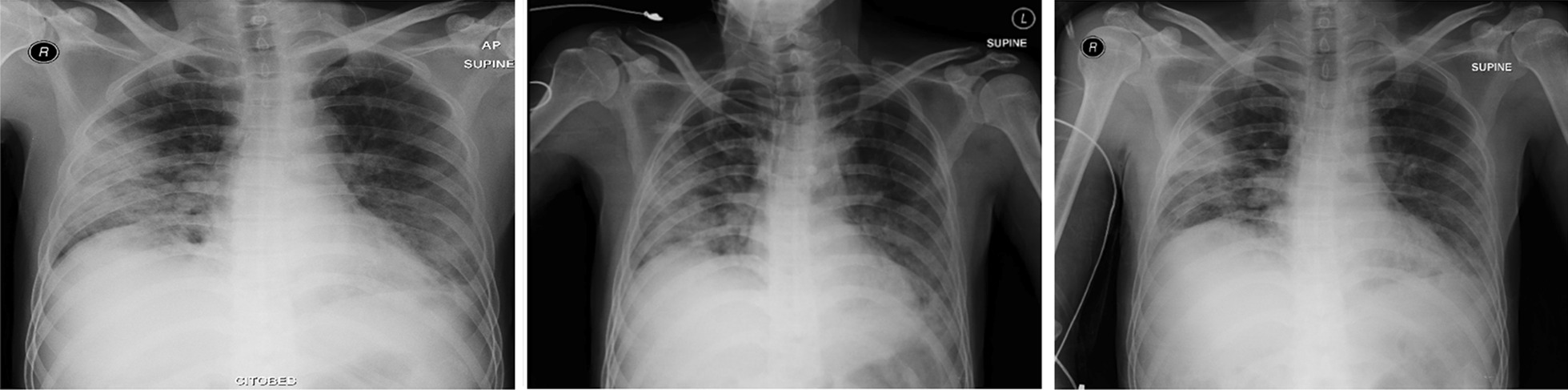
Thorax radiology image of patient 1 before the first CPT on 6 August 2020 (left), before the second CPT on 9 August 2020 (middle) and 5 days after the second CPT on 14 August 2020 (right)

**Fig. 2:**
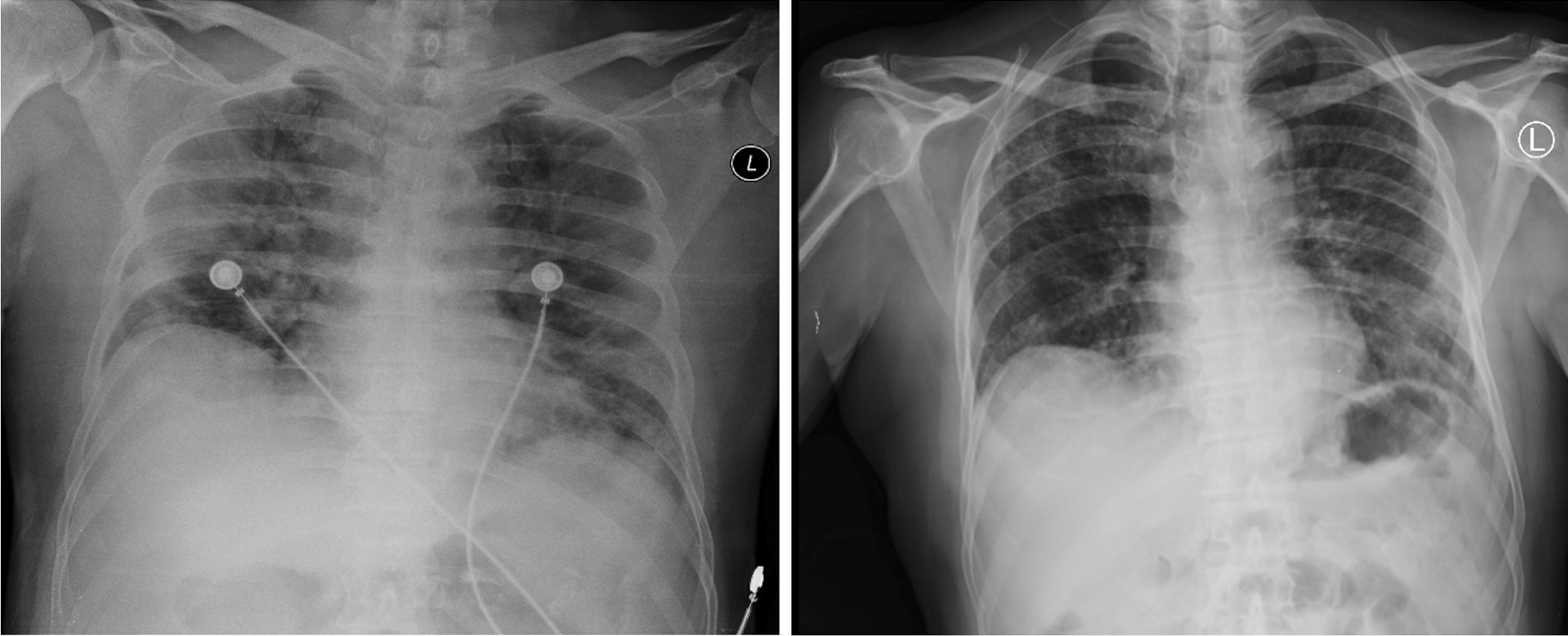
Thorax radiology image of patient 2, 6 days before CPT on 4 November 2020 (left) and 12 days after second CPT on 23 November 2020 (right)

**Fig. 3:**
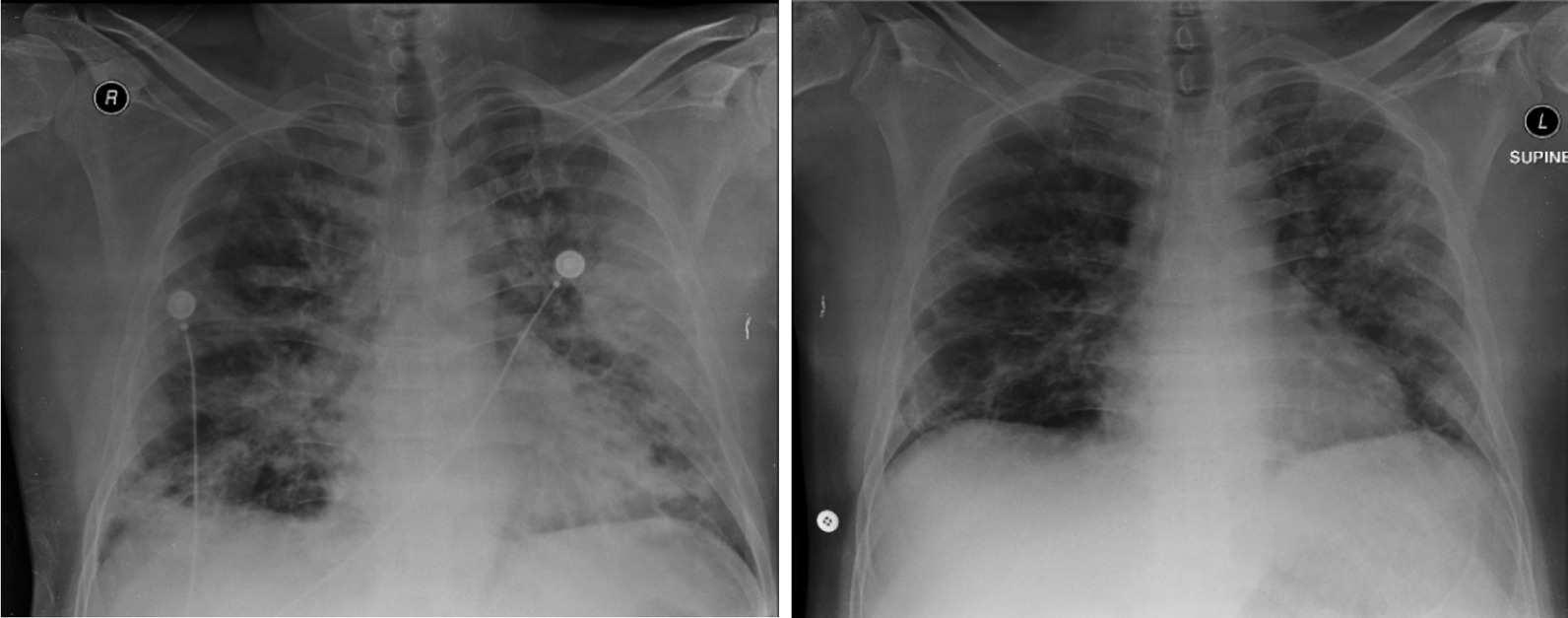
Thorax radiology image of patient 3, 1 day before the first CPT on 5 September 2020 (left) and 11 days after the second CPT on 20 September 2020 (right)

**Fig. 4:**
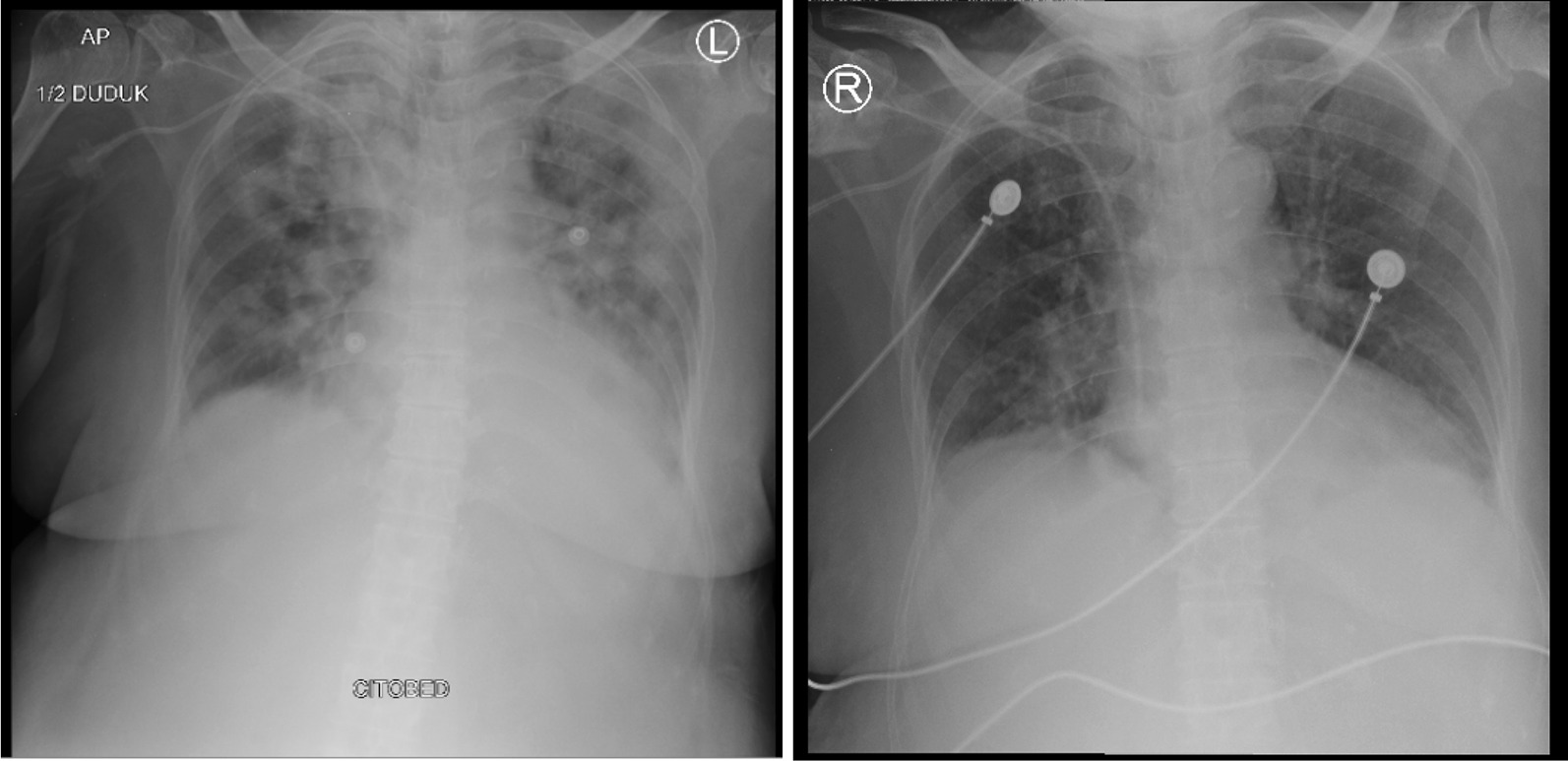
Thorax radiology image of patient 8, 2 days before the first CPT on 19 December 2020 (left) and 6 days after the second CPT on 27 December 2020 (right)

All patients showed bilateral suprahilar, perihilar, and paracardial pulmonary parenchymal infiltrates and consolidation on thorax imaging. Only one patient had a thorax CT scan, which showed GGO. The limited CT scanning primarily depended on financial ability and insurance coverage. Improvement occurred 1 week after CPT, and the healing process was more obvious within or after a second week after CPT. Remarkable recovery can be seen in Fig. 1 (patient 1), Fig. 2 (patient 2), Fig. 3 (patient 3), and Fig. 4 (patient 8).

The specific features of laboratory parameters in COVID-19 patients are lymphocytopenia, increased C-reactive protein (CRP) levels, and decreased liver function. All patients showed CRP levels above normal level with median CRP was 28.975 (ranging from 15.6 to 72.0) before CP administration, but only two patients had serial CRP value monitoring, and they showed a lower CRP level after CPT (patients 4 and 7). The same issues with thoracic CT scans occurred for laboratory examinations, as well as efforts to reduce reagent consumption and the workload of hospital laborers because of the increasing number of COVID-19 patients.

The patients with two doses of CP tended to have faster recovery than patients with one dose of CP, including fewer mean number of days with a decreased respiratory rate (1.5 days versus 2.75 days) and with decreased oxygen supplementation (3 days versus 3.75 days), but both groups had the same mean number of days with increased oxygen saturation (2 days).

At the time of CP transfusion, all patients showed positive RT-PCR results. Following CPT, the RT-PCR results were negative for all four patients with two doses of CP, two patients with one dose of CP tested negative, and two patients showed an obvious increase in CT value, even though the result was still positive. These results support a neutralizing effect of antibodies in plasma against SARS-CoV-2 and a sufficient amount of antibody needed to eliminate the virus. The PCR results are presented in Table 4.

**Table 4:** Positive-to-negative conversion in patients receiving Convalescent Plasma Therapy

Patient	CPT date		Before CPT			After CPT		
	First	Second	Date	RT-PCR	Ct value	Date	RT-PCR	Ct value
1	6 August 2020	9 August 2020	3 August 2020	Positive	Ct value: 24.54	25 August 2020	Negative	
2	10 November 2020	11 November 2020	31 October 2020	Positive	RDRP gene: 21.77	2 December 2020	Negative	
3	6 September 2020	9 September 2020	2 September 2020	Positive	CT value: 31.00	29 September 2020	Negative	
4	24 December 2020	30 December 2020	16 December 2020	Positive	RDRP gene: 21.11	2 January 2020	Negative	
5	10 November 2020	–	5 November 2020	Positive	RDRP gene: 28.56	30 November 2020	Negative	
6	12 November 2020	–	5 November 2020	Positive	RDRP gene: 33.13	15 November 2020	Positive	RDRP gene: 39.70
7	22 August 2020	–	21 August 2020	Positive	Ct value: 22.62	21 September 2020	Negative	
8	21 December 2020	–	13 December 2020	Positive	RDRP gene: 25.28	24 December 2020	Positive	RDRP gene: 35.93

RT-PCR: Real time polymerase chain reaction, CT Value: Cycle threshold value, RDRP gene: RNA-dependent RNA polymerase gene

## Discussion

Our study is the first case series in Indonesia to explore the feasibility of CPT in eight non-intubated COVID-19 patients. One to two doses of 200 mL CP were tolerated well, followed by a significant improvement in clinical symptoms within 1–7 days after the first CP administration.

This study showed that CPT within 14 days after symptom onset and within a week after patient admission tends to prevent disease progression. The improvement occurred soon after CP administration, especially in patients who received two doses of CP. Based on this situation, there are three important factors that influence the success of CPT [7–9].

First is the timing of CPT. The lung is the first and main target organ affected in COVID-19; dysfunctional respiration accompanied by rapid viral replication leads to massive inflammatory cell infiltration and proinflammatory cytokine production, resulting in cytokine storms in lung alveoli as the body attempts to eliminate the virus. When this effort fails, the excessive levels of released cytokines cause acute respiratory distress syndrome (ARDS), other organ damage, hypercoagulability, and ultimately death. Mortality in COVID-19 is not directly caused by the virus but by the side effects of excessive cytokine production as a reaction to virus presentation [7].

The best time for CPT is during viral replication and before a cytokine storm occurs. The antibodies contained in the CP mainly function to eliminate SARS-CoV-2 but not to repair organ damage resulting from cytokine storms. Convalescent plasma can also modulate the inflammatory reaction, but this activity is suspected in only a limited number of cases. When the antibodies succeed in eradicating the virus, the impending cytokine storm is prevented.

However, what if the cytokine storm has already started? There is still a place for CPT as long the PCR result is positive even if the cytokine storm has already occurred. As described previously, abundant inflammatory mediators are released during cytokine storms to eliminate the virus. When the viral load is reduced by CP, the inflammatory reaction diminishes, as does mediator production. Comorbid conditions make the effective period narrower. Based on the viral load and clinical symptom output, the best time for CPT in patients with comorbid conditions is within the first week of fever onset or the first 72 hours after the occurrence of dyspnea [9–11].

Better outcomes have been observed in SARS patients given plasma within the first 14 days than in those treated after 14 days (58.3% versus 15.6%; *p* < 0.01). Some studies have even suggested a shorter period of 1 week. This recommendation is consistent with viral load and shedding durations. The viral load in mild patients showed a significant reduction by 14 days, but the virus load was still high after 14 days in the severe condition. Viral shedding in survivors ended within 20–21 days, but the process occurred continually in non-survivors. Earlier application of CPT will shorten the durations of high viral load and shedding, resulting in faster positive-to-negative conversion of RT-PCR tests [12, 13].

All patients in this study received their first dose of CPT within 14 days after the first symptoms occurred, with a median time of 13 days. Most of the patients had at least one comorbid condition, including age, and the median time from admission to the first CPT was 6.5 days. The improvement occurred, at the earliest, 1 day after CPT, as the antibodies started to work soon as they entered the patient’s body.

The second and third important factors investigated were the sufficient plasma dose and antibody levels. The first optimal dose of CP is one bag (200 mL) with a 1:320 antibody level. One study reported that the dose of plasma was 3–5 mL/kg body weight (BW) for adults and 10 mL/kg BW for pediatric patients. Convalescent plasma therapy can be repeated within 48 hours, depending on the clinical and laboratory conditions. Two doses of CP tended to yield better and faster recovery than one dose of CP, including fewer days with a decreased respiratory rate (the average days were 1.5 versus 2.75 days) and decreased oxygen supplementation (the average days were 3 versus 3.75 days). The antibody level in recovered COVID-19 donors stayed at the maximal value within 4 months and then decreased gradually, reaching undetectable levels in 25.6% (IgG) and 16.1% (neutralizing antibodies) of patients at 36 months after disease onset. These data suggest that neutralizing antibodies in plasma from recently recovered patients should be effective against the virus [7, 14, 15].

There were some limitations to this study. First, in addition to CPT, all patients received standard treatment, including antiviral treatment, despite the uncertainty of the drug’s efficacy. Patients were also treated with corticosteroids, which may influence the immune response and delay virus clearance. Second, the antibody level before and after CPT was not measured; therefore, it should be further clarified. Third, the limited laboratory, pulmonary radiology, and functional examinations were mainly based on limited insurance coverage and efficiency efforts to reduce reagent consumption and the workload of hospital laborers because of the increasing number of COVID-19 patients. The results of this pilot case study are very promising, but it was not a randomized placebo-controlled trial, and such studies are warranted in the future.

## Conclusion

This pilot study showed a potential effect of CP in the treatment of non-intubated COVID-19 patients. One to two doses of CP with at least 1:320 antibody levels within 14 days within initial symptom onset can rapidly reduce the viral load and improve the clinical outcome; patients with two doses of CP tend to have better and faster recovery than patients with one dose of CP. Further randomized controlled trials with larger patient cohorts are needed to confirm the definite clinical benefits of CPT in COVID-19 patients.

## Data Availability

The dataset and materials from this study are available from the corresponding author.
